# Reprogrammable CRISPR/Cas9-based system for inducing site-specific DNA methylation

**DOI:** 10.1242/bio.019067

**Published:** 2016-05-11

**Authors:** James I. McDonald, Hamza Celik, Lisa E. Rois, Gregory Fishberger, Tolison Fowler, Ryan Rees, Ashley Kramer, Andrew Martens, John R. Edwards, Grant A. Challen

**Affiliations:** 1 Center for Pharmacogenomics, Department of Medicine, Washington University in St. Louis School of Medicine, St. Louis, MO, USA; 2 Section of Stem Cell Biology, Division of Oncology, Department of Medicine, Washington University in St. Louis School of Medicine, St. Louis, MO 63110, USA; 3 College of Arts and Science, Washington University in St. Louis, St. Louis, MO 63130, USA; 4 Developmental, Regenerative and Stem Cell Biology Program, Division of Biology and Biomedical Sciences, Washington University in St. Louis School of Medicine, St. Louis, MO 63110, USA

**Keywords:** CRISPR/Cas9-based system, CpG dinucleotides, DNA methylation

## Abstract

Advances in sequencing technology allow researchers to map genome-wide changes in DNA methylation in development and disease. However, there is a lack of experimental tools to site-specifically manipulate DNA methylation to discern the functional consequences. We developed a CRISPR/Cas9 DNA methyltransferase 3A (DNMT3A) fusion to induce DNA methylation at specific loci in the genome. We induced DNA methylation at up to 50% of alleles for targeted CpG dinucleotides. DNA methylation levels peaked within 50 bp of the short guide RNA (sgRNA) binding site and between pairs of sgRNAs. We used our approach to target methylation across the entire CpG island at the *CDKN2A* promoter, three CpG dinucleotides at the *ARF* promoter, and the CpG island within the *Cdkn1a* promoter to decrease expression of the target gene. These tools permit mechanistic studies of DNA methylation and its role in guiding molecular processes that determine cellular fate.

## Abstract (precis)

**Summary:** We developed a CRISPR/dCas9-DNMT3A fusion protein to repress the expression of endogenous genes in combination with multiple guide RNAs. This tool can help us elucidate the role of DNA methylation in normal development and disease.

## INTRODUCTION

DNA methylation of CpG dinucleotides is a prominent epigenetic modification of the mammalian genome that can influence gene expression, and aberrant distribution of DNA methylation is associated with a spectrum of human disorders including cancers (Egger et al., 2004). Despite intensive study, it remains unclear which CpG dinucleotides must change methylation state in order to alter transcription. Genome-wide analyses have found associations between DNA methylation and reduced gene expression that occur both in the proximal promoter and downstream of the gene's transcription start site (TSS) (Bell et al., 2011; Bock et al., 2012; Lou et al., 2014; VanderKraats et al., 2013; Lund et al., 2014). However, evidence supports both that DNA methylation can cause a loss of expression, and that expression changes can alter DNA methylation patterns (Bestor et al., 2015; Busslinger et al., 1983). Here, we sought to develop tools for locus-specific epigenetic remodeling to directly address the role of DNA methylation in regulating gene expression.

Targeted DNA methylation approaches have been attempted by fusing DNA methyltransferase enzymes (DNMTs) to DNA-binding proteins such as zinc finger proteins (ZFPs) (Siddique et al., 2013), and transcriptional activator-like effector (TALE) (Bernstein et al., 2015). However, engineering custom proteins for each targeted sequence is laborious and requires specialized expertise. Moreover, in these studies, induced DNA methylation of the targeted loci was relatively poor, with substantial off-target activity. An engineered form of the clustered, regularly interspaced, short palindromic repeat (CRISPR) system has emerged as an alternative for achieving site-specific DNA targeting (Jinek et al., 2012). Here, the Cas9 endonuclease is directed to genomic targets by engineered short guide RNAs (sgRNAs) (Jinek et al., 2012). Because the sgRNA is the DNA sequence-specific component of the system, it allows for efficient targeting of multiple regions due to the ease of design and synthesis of new sgRNAs (relative to engineering new custom proteins for each target site). A Cas9 mutant (D10A and H840A; henceforth referred to as dCas9) that lacks endonuclease activity but can still be recruited by sgRNA(s) (Jinek et al., 2012) has recently been used to target genes in mammalian cells for transcriptional activation (Perez-Pinera et al., 2013a,b; Maeder et al., 2013; Mali et al., 2013). Here, we demonstrate an easily reprogrammable CRISPR/dCas9 DNMT fusion capable of inducing site-specific DNA methylation.

## RESULTS

**Fig. 1.**
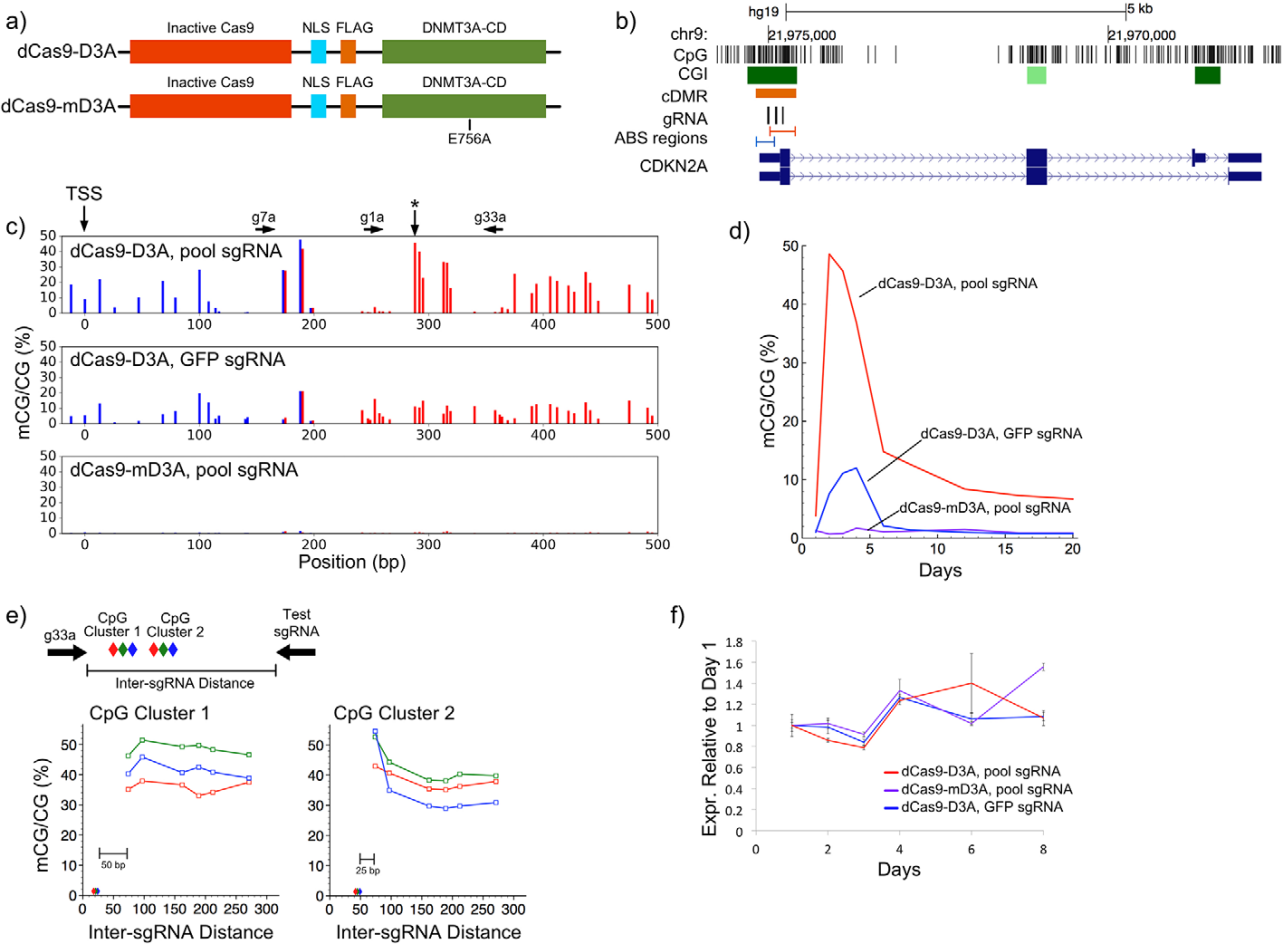
**Site-specific induction of DNA methylation using a CRISPR-Cas9 DNMT fusion.** (A) dCas9-DNMT3A CD fusion constructs. The E756A mutation inactivates the DNMT3A-CD. NLS, nuclear localization signal; FLAG, FLAG tag domain. (B) UCSC genome browser view showing the locations of the three *CDKN2A* sgRNA (g1a, g7a and g33a). The three sgRNA were validated to ensure they targeted this locus (Fig. S1.2). cDMR indicates the region from the literature where methylation changes are associated with expression changes (Fig. S1.1). (C) Induced DNA methylation at the *CDKN2A* promoter three days post-transfection. Colors correspond to the red and blue ABS regions in B. Three CpGs were independently measured in both amplicons. sgRNA target sites are indicated above the graphs. Pool sgRNA indicates g1a, g7a, g33a were used simultaneously. Sanger sequencing validation is presented in Fig. S1.3, and non-CpG methylation data is presented in Fig. S1.4. (D) Time course of the percent methylation data for the CpG marked with an asterisk in C. Additional CpGs are shown in Fig. S1.5. (E) Methylation induced by a pair of sgRNA decreases with increasing intervening distance. Distance is calculated relative to the 3′ end of the g33a sgRNA. Diamonds indicate the location each CpG monitored for methylation; whose color corresponds to appropriate line in the graph. Additional data from individual and paired sgRNA is presented in Fig. S1.6. (F) *CDKN2A* expression for samples with induced methylation. Expression is normalized to day one for each respective sample. Error bars=mean±s.e.m. (*n*=1 performed in technical triplicate).

To design a flexible system to target DNA methylation, we fused dCas9 to the catalytic domain of the *de novo* DNA methyltransferase DNMT3A (Fig. 1A). To test this system we targeted DNA methylation to the tumor suppressor gene *CDKN2A* (cyclin dependent kinase 2A), which inhibits progression through the cell cycle (Liggett and Sidransky, 1998). *CDKN2A* is one of the most frequently hypermethylated genes in The Cancer Genome Atlas (Ciriello et al., 2013), and numerous clinical studies show a negative correlation between *CDKN2A* methylation and expression in colorectal cancer (Shima et al., 2011). While it is generally assumed that *CDKN2A* methylation induces gene silencing, it has also been suggested that DNA methylation occurs after the loss of expression (Hinshelwood et al., 2009). From a literature search, we identified 17 publications that associate *CDKN2A* methylation with expression and/or cancer (Fig. S1.1). Overwhelmingly, these papers studied the differentially methylated region (cancer DMR, cDMR) on the 3′ end of the CpG island that overlapped the first exon of *CDKN2A* (Fig. 1B).

We computationally designed three sgRNAs (g1a, g7a and g33a) to target this region and test whether DNA methylation was sufficient to induce gene silencing. We validated the ability of each sgRNA to target the *CDKN2A* locus by transfecting them with active CRISPR/Cas9 and measuring the ability of Cas9 to cleave the locus (Fig. S1.2, Table S1). We then transfected HEK293T cells with the pool of three sgRNAs along with either a normal dCas9-DNMT3A catalytic domain (CD) fusion (dCas9-D3A) or one with a DNMT3A^E756A^ mutation (dCas9-mD3A, Fig. 1A), which abolishes DNA methyltransferase activity (Reither et al., 2003). Transfection efficiencies were >80-90% for all experiments as measured by co-transfection with GFP-containing plasmids. We analyzed DNA methylation levels for 20 days post-transfection using Illumina sequencing of two amplicon regions (amplicon bisulfite sequencing, ABS). ABS results were validated using Sanger bisulfite sequencing (R^2^=0.83; Fig. S1.3), and DNA methylation levels at CpGs analyzed in both of two independent ABS amplicons showed strong correspondence (R^2^=0.98; Fig. 1C). All CpGs had >100× sequencing coverage, with a median coverage of 15,200.

Over the 20-day time course we observed an increase in DNA methylation at the *CDKN2A* target locus that ranged from 20-43% at its peak on day three. Background methylation from transfection with dCas9-mD3a was consistently less than 1.5%, while background methylation from an off-target sgRNA, which controls for DNMT3A-CD overexpression, was less than 14% on day three. Induced DNA methylation levels were highest over a set of eight CpGs directly between the g33a and g7a sgRNA target sites (Fig. 1C). Increases in CHG and CHH methylation were minimal (Fig. S1.4). DNA methylation decreased rapidly after passaging the cells on day four, but stabilized 20 days post-transfection at 6-10% (Fig. 1D; Fig. S1.5). Despite the literature support for a negative correlation between expression and DNA methylation in this region, we did not observe a measurable effect on *CDKN2A* gene expression by RT-qPCR (Fig. 1F). This suggests that a limited increase in methylation in the region 100-400 bp downstream of the *CDKN2A* TSS is insufficient to trigger gene silencing.

Spatially, the induced DNA methylation spiked near the sgRNA target sites and dropped quickly toward background levels at surrounding CpG sites. Analysis of DNA methylation induced by single sgRNAs indicates that methylation occurs primarily within 50 bp of the sgRNA binding site (Fig. S1.6). Higher DNA methylation levels were often observed 3′ of the sgRNA binding site (Fig. S1.6). Our initial data from the three pooled sgRNAs suggested that CpG methylation was higher between pairs of sgRNAs. To investigate this effect, we transfected pairs of sgRNA with varying intervening distances and monitored methylation of six clustered CpGs between the sgRNA pairs using ABS (Fig. 1E). DNA methylation of the three CpGs (cluster 1) within 20 bp of the fixed sgRNA (g33a) did not change with addition of a second sgRNA 77 bp or further away. However, the methylation level increased from 30-40% to 42-53% when sgRNAs were paired within 80 bp and both sgRNAs were within 50 bp of CpG cluster 2 (Fig. 1E). This suggests the DNMT3A-CD activity at the target locus is additive.

**Fig. 2.**
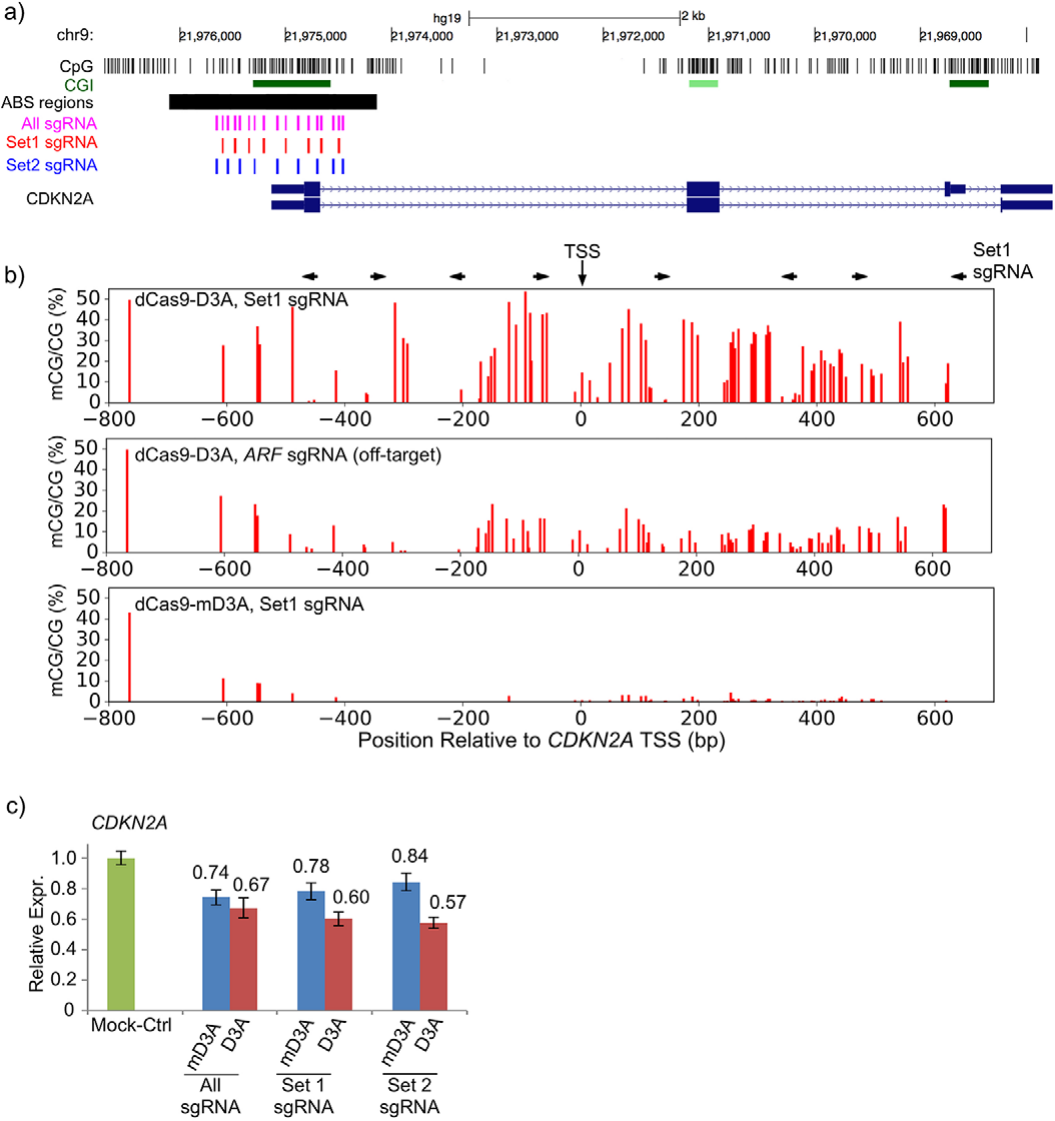
**DNA methylation decreases gene expression at the *CDKN2A* promoter in a context dependent manner.** (A) Locations of the 17 sgRNAs used in Sets 1 and 2 and regions sequenced in B. sgRNA coordinates are in Table S1. (B) Percent methylation is plotted for regions designated in A. Set 1 sgRNA target sites are indicated above the graphs. Data for All sgRNA, Set 2 sgRNA, and off-target SgRNA is presented in Fig. S2. (C) Methylation induced by *CDKN2A*-targeted sgRNA Set 1, Set 2, and All sgRNA, decreases gene expression. Relative expression of *CDKN2A* is normalized to a mock-treated control. Error bars=mean±s.e.m. (All, *n*=2; Set 1, *n*=1; Set 2, *n*=1; all performed in technical triplicate, paired *t*-test).

We next tested whether we could use our approach to methylate an entire CpG-island (CGI). We designed 17 sgRNAs (Fig. 2A; Table S1) to target DNA methylation across the *CDKN2A* CGI, which spans the TSS. We applied three combinations of sgRNAs (Set 1, 2, All) to test whether inducing DNA methylation of the entire CGI could decrease gene expression (Fig. 2A). ABS analysis of eight amplicons (minimum per CpG sequencing depth of 100) showed that the DNA methylation level increased to an average of 22% across the entire region with a peak of 54% (Fig. 2B). As an off-target negative control, we used three sgRNAs targeted to the *ARF* promoter located ∼20 kb away. The average background methylation at *CDKN2A* after treatment with off-target *ARF* sgRNAs was 9% (Fig. 2B). The other two sgRNA sets (Set 2 and All) induced similar increases of methylation across the CGI overlapping the TSS of *CDKN2A* (Fig. S2).

Analysis of *CDKN2A* expression by RT-qPCR in all three sgRNA targeting experiments (Set 1, 2, All) indicated an average 39% decrease in *CDKN2A* mRNA expression after targeting with dCas9-D3A (Fig. 2C). Cells transfected with dCas9-mD3A showed a 16-26% reduction in *CDKN2A* expression, likely due to CRISPR inhibition (Fig. 2C). Across the three replicates (Set 1, 2, All) expression decreased by an average of 17% in dCas9-D3A relative to dCas9-mD3A (*P*<0.01 paired-*t*-test). This indicated that DNA methylation directly decreased *CDKN2A* expression, but targeting of the entire CGI was required to trigger this effect. Our results are consistent with other studies that find a similar reduction in gene expression after inducing methylation at the *CDKN2A* promoter using ZFP- and TALE-based systems (Bernstein et al., 2015; Cui et al., 2015).

**Fig. 3.**
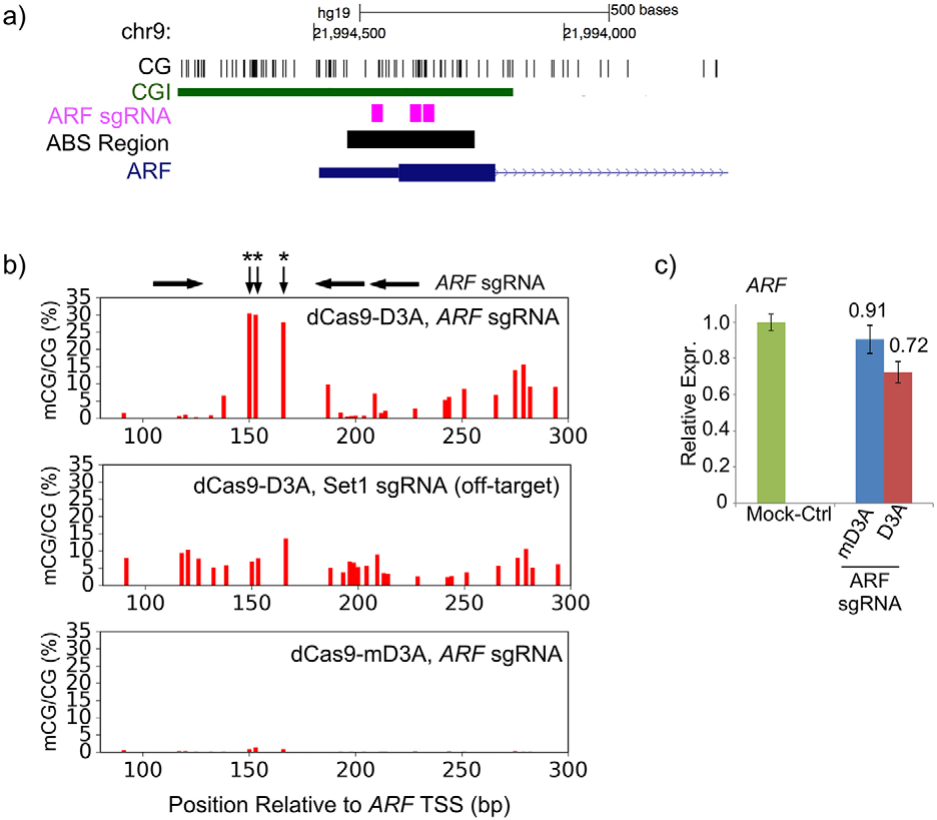
**Induced methylation decreases *ARF* expression.** (A) Locations of the three sgRNA used to induce DNA methylation at the *ARF* promoter. (B) Percent methylation is plotted for a region of exon 1 of the *ARF* locus. *ARF* sgRNA target sites and three targeted CpGs (asterisks) are indicated above. (C) Methylation induced by *ARF* targeted sgRNA decreases gene expression. Relative expression of *ARF* is normalized to a mock-treated control. Error bars=mean±s.e.m. (*n*=1 biological replicate performed in technical triplicate).

To verify the effect of our system with a separate locus, we designed three inwardly-directed (5′ to 3′) sgRNAs that bracketed three CpG sites in the *ARF* promoter located 150-170 bp downstream of the TSS (Fig. 3A). *ARF*-targeted sgRNAs increased the DNA methylation level to 27-30% at these three CpG sites with less than 15% methylation induced in adjacent sites (Fig. 3B). Induced methylation of the *ARF* promoter was associated with a 19% decrease in its expression (Fig. 3C).

**Fig. 4.**
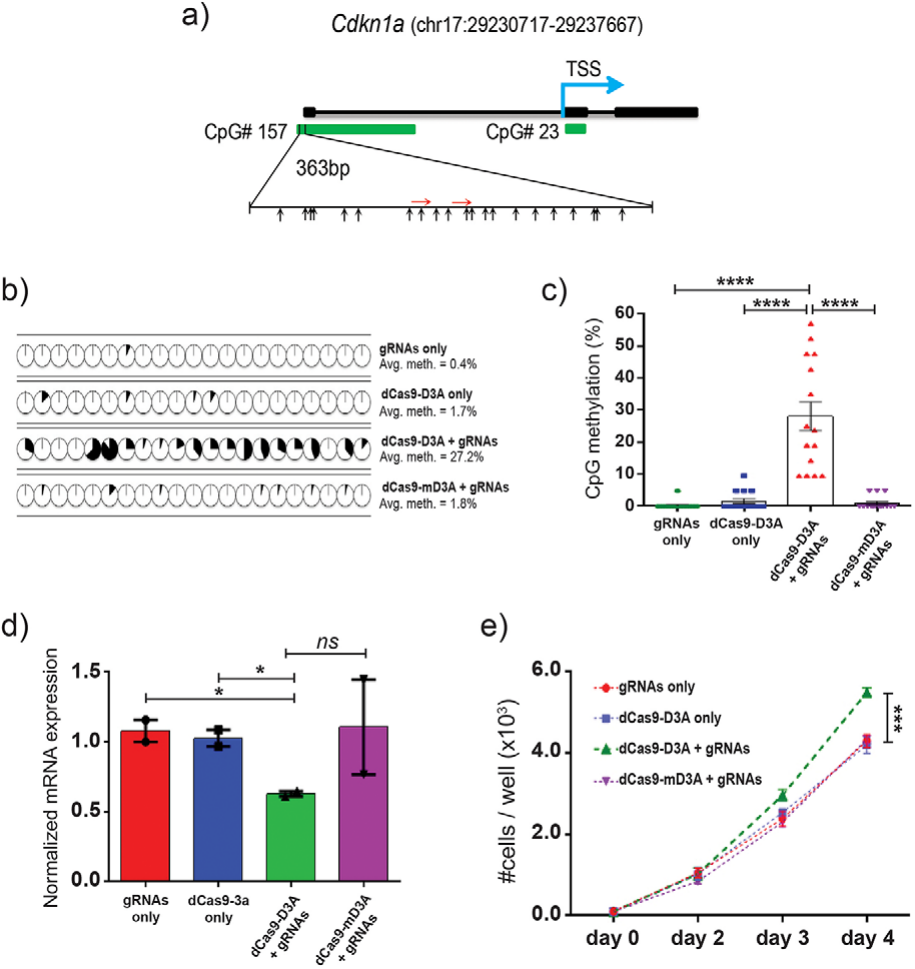
**Targeted CpG methylation of the *Cdkn1a* locus using dCas9-D3A fusion protein.** (A) Organization of the mouse *Cdkn1a* target locus with CpG islands indicated with green boxes. Blue arrow indicates the location of transcriptional start site (TSS). Red arrows indicate the relative position of sgRNA binding sites (5′ to 3′) within the 5′ CpG island. Black arrows indicate the relative positions of CpGs. (B) Bisulfite sequencing analysis of *Cdkn1a* promoter in 32D cells transduced with sgRNAs or dCas9-D3A only, and dCas9a-D3A or dCas9-mD3A with sgRNAs. Changes in methylation of specific CpGs are indicated by pie charts. Shading indicates the average amount of DNA methylation at each CpG (*n*=14-15 biological replicates representative of three independent experiments). (C) Average DNA methylation level of target locus within *Cdkn1a* promoter (unpaired *t*-test). (D) mRNA expression level of *Cdkn1a* (normalized to 18S) in transduced 32D cells (*n*=2 biological replicates performed in technical duplicate; unpaired *t*-test). (E) Growth kinetics of transduced 32D cells (*n*=2 biological replicates performed in technical quadruplicate; unpaired *t*-test). *P<0.05, ****P<0.0001, error bars=mean±s.e.m.

To further validate our design rules, we attempted to induce DNA methylation in a different system in a regulated fashion with a minimalistic combination of sgRNAs. dCas9-D3A and dCas9-mD3A were cloned into a doxycycline-inducible lentivirus where expression of the fusion protein is linked to mCherry via internal ribosome entry site (IRES), and targeted to the *Cdkn1a* locus with two rationally-designed sgRNAs. We designed two sgRNAs targeting the CpG island located in the *Cdkn1a* promoter (Fig. 4A) and cloned them into lentiviral vectors (in which sgRNAs and GFP are expressed constitutively from two different promoters) (Fig. S4.1) and transduced 32D cells (mouse IL3-dependent myeloid progenitor cell line with an unmethylated *Cdkn1a* promoter and active *Cdkn1a* expression). Additional negative controls were generated by transducing 32D cells with sgRNAs only, dCas9-D3A only, or sgRNAs plus dCas9-mD3A. Cells were then FACS-sorted by GFP and/or mCherry to establish stable cell lines. Analysis of the stable cell lines induced by doxycycline showed successful expression of dCas9-fusions and sgRNAs via mCherry and GFP signal (Fig. S4.2). In the absence of doxycycline, the expression of fusion constructs was silenced (Fig. S4.2).

Stable cells were induced with doxycycline for eight days. *Cdkn1a* promoter DNA methylation and gene expression were then evaluated by bisulfite sequencing and RT-qPCR respectively. Cells transduced with dCas9-D3A together with sgRNAs showed an increase in *Cdkn1a* promoter DNA methylation by over 25% across the entire target region (and by as much as 40-85% at individual CpGs around sgRNAs) compared to all control groups (Fig. 4B,C), and a reduction in *Cdkn1a* expression by approximately 40% (Fig. 4D). There was no difference in *Cdkn1a* promoter DNA methylation or expression between any control groups (Fig. 4B-D). Cells transduced with dCas9-D3A together with sgRNAs showed a growth advantage, consistent with an increase in proliferation due to repression of a cell cycle inhibitor (Fig. 4E). As with targeting of the human *CDKN2A* locus, these data suggested that induced DNA methylation pioneered by dCas9-D3A targeted with a minimal combination of sgRNAs can influence gene expression and generate subsequent downstream functional phenotypes.

**Fig. 5.**
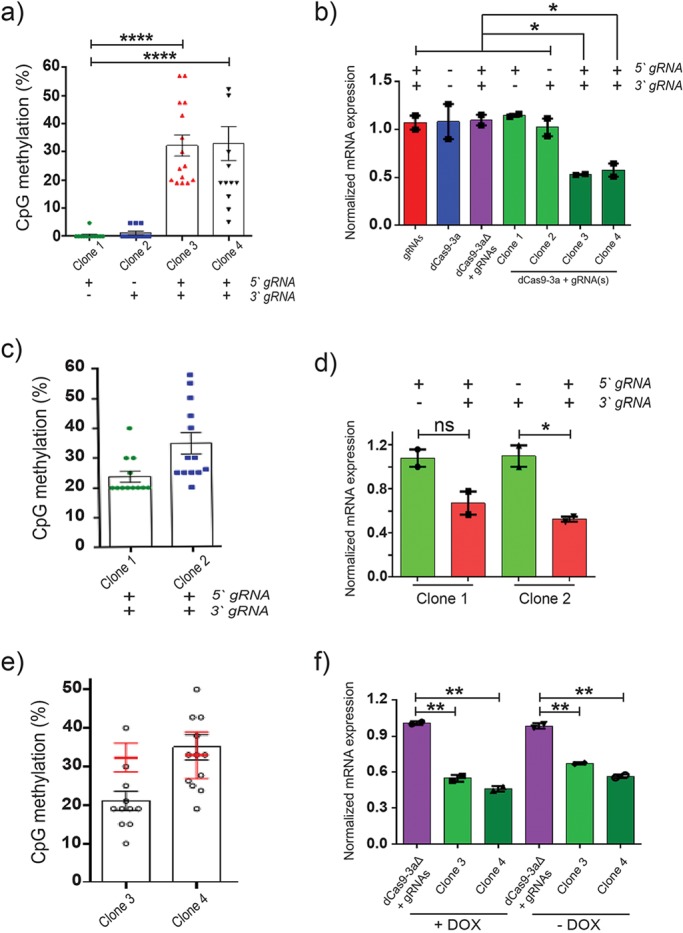
**Multiple sgRNAs are required for the efficient establishment of targeted methylation.** (A) Average DNA methylation level of target locus within *Cdkn1a* promoter (*n*=12-15 biological replicates representative of two independent experiments; unpaired *t*-test). Presence or absence of each sgRNA in each clone is shown (−; +) at the bottom of the graph. (B) Expression level of *Cdkn1a* mRNA in 32D cells transduced with sgRNAs, dCas9-D3A, dCas9-mD3A with sgRNAs or dCas9-D3A with either one or both sgRNAs (*n*=2 biological replicates performed in technical duplicate; unpaired *t*-test). Presence or absence of each sgRNA in each clone is shown (−; +). (C) Average promoter DNA methylation (*n*=12-15 biological replicates representative of two independent experiments) or (D) expression level of *Cdkn1a* in 32D cells in 32D cells with one sgRNA which were then transduced with their complementary sgRNA (*n*=2 biological replicates performed in technical duplicate; unpaired *t*-test). (E) Average DNA methylation of *Cdkn1a* target locus for clones following doxycycline withdrawal for eight days. Clones were previously induced for 12 days, with starting induced DNA methylation level shown by red error bars (*n*=12-16 biological replicates representative of two independent experiments). (F) Expression level of *Cdkn1a* in clones after eight days of de-induction (*n*=2 biological replicates performed in technical duplicate; unpaired *t*-test). *P<0.05; **P<0.01; ****P<0.0001, error bars=mean±s.e.m.

We reverse cloned viral integrants to identify clones that contained either one of the two guides, or both sgRNAs together with dCas9-D3A. No induced promoter DNA methylation or reduction in gene expression was observed in clones expressing only a single sgRNA (Fig. 5A,B). In contrast, *Cdkn1a* promoter methylation was increased and its expression decreased in clones that expressed both sgRNAs (Fig. 5A,B). To confirm the presence of both sgRNAs were required to execute induced DNA methylation, clones that contained only a single sgRNA were transduced with their complementary sgRNA. Following this, after eight-days of induction, *Cdkn1a* DNA methylation increased across the promoter by up to 33% compared to the parental clone that contained only a single sgRNA (Fig. 5C). *Cdkn1a* expression was reduced by 40-50% in the complemented clones (Fig. 5D). These data indicate that targeting with multiple sgRNAs results in a synergism and induces robust DNA methylation. We also examined the stability of the induced DNA methylation by withdrawing doxycycline for eight days. *Cdkn1a* promoter DNA methylation and expression profiles remained largely unchanged (Fig. 5E,F) even though expression of dCas9-D3A was silenced (Fig. S4.2).

**Fig. 6.**
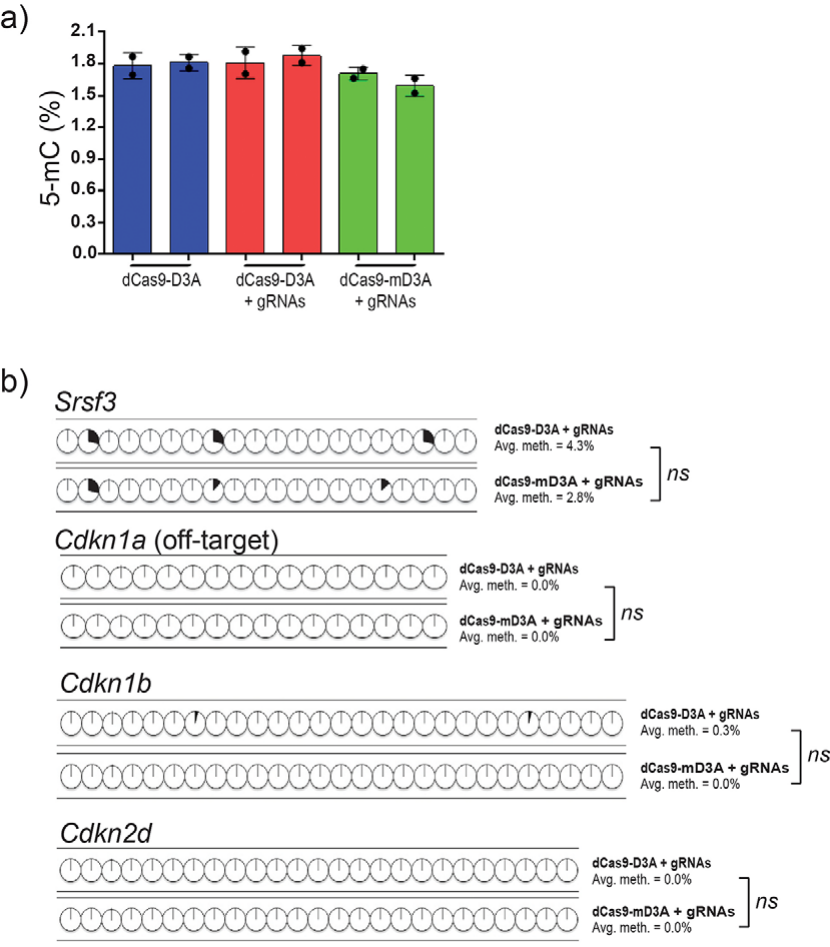
**Limited off-target activity in dCas9-D3A driven targeted DNA methylation system.** (A) Global 5-mC content levels in cells transduced with only dCas9-D3A, dCas9-D3A with sgRNAs, or dCas9-mD3A with sgRNAs targeting *Cdkn1a* locus (*n*=2 biological replicates performed in technical duplicate; unpaired *t*-test). (B) Bisulfite sequencing analysis of off-target CpG islands located upstream or downstream of *Cdkn1a* target locus (*Srsf3* and off-target *Cdkn1a*, respectively) or distant loci (*Cdkn1b* and *Cdkn2d*) in cells that are transduced with dCas9-3a or dCas9-3a with both sgRNAs (*n*=10-24). Source data in Table S5. Error bars=mean±s.e.m.

To assess non-specific activity in this system, we initially attempted to analyze off-target genomic areas with high homology to predicted binding regions of both *Cdkn1a* sgRNAs. However, no regions with high homology were found as these sgRNAs were designed based on their minimal off-target binding and the presence of a PAM-site. As an alternative, we measured the global DNA methylation levels between cells that contained fusion proteins (dCas9-D3A or dCas9-mD3A) in the presence of both sgRNAs (Fig. 6A), as well as the local DNA methylation profile of CpG islands located upstream and downstream of the *Cdkn1a* target locus (*Srsf3* and off-target *Cdkn1a* respectively), and the promoter CpG islands of the related cell cycle family members *Cdkn1b* and *Cdkn2d* (Fig. S6). *Srsf3* and off-target *Cdkn1a* are adjacent to *Cdkn1a* target locus on chromosome 17 (chr17), whereas *Cdkn1b* (chr6) and *Cdkn2d* (chr4) are distant loci. We found no statistically significant difference in global 5-methylcytosine (5-mC) levels between different groups or across the regions that were analyzed (Fig. 6A,B).

## DISCUSSION

We provide an outline for using a modified CRISPR/dCas9 system to evaluate the functional relevance of DNA methylation at specific CpGs and described guidelines for its use. DNA methylation induction occurs within ∼50 bp of a sgRNA target site and is strongest between two adjacent and inwardly directed sgRNA binding sites. Based on our design criteria, we designed sets of sgRNAs that induced methylation at the human *CDKN2A* and *ARF* promoters, and the mouse *Cdkn1a* promoters with similar efficiency. Induced methylation was sufficient to decrease expression of all three genes. Methylation increases and changes in expression were highly significant and reproducible either by using multiple distinct sgRNA combinations in the case of *CDKN2A* or at the clonal levels as observed for *Cdkn1a*. Moreover, the reduction in *Cdkn1a* expression clearly had functional consequences (increased proliferation) for the transduced cells. Though modest, the expression decreases caused by induced methylation are consistent with previously published results using ZFP and TALE fusions (Bernstein et al., 2015; Cui et al., 2015).

The effects of the induced methylation also appeared to be context dependent. While methylation of the entire CGI at the *CDKN2A* promoter repressed gene expression, inducing DNA methylation of a region 100-400 bp downstream of the *CDKN2A* TSS alone was insufficient to affect expression despite the frequent observation of a negative correlation between methylation and expression in this region. This indicates the importance of the flexibility to target multiple regions offered by our CRISPR/dCas9 DNMT fusion system.

Although approaches for targeted DNA methylation have been previously described, our method is advantageous for several reasons including; (1) the ease of designing new sgRNAs for targeting, (2) higher levels of induced DNA methylation, (3) little off-target activity. This approach can be used to interrogate the effects of DNA methylation on only a few CpG sites by bracketing them with sgRNAs, or can be used to test the effects of broader increases in DNA methylation by using many sgRNAs simultaneously. Further, the method described here provides a robust, reprogrammable approach to allow researchers to easily and thoroughly explore the functional roles of DNA methylation changes in development and disease.

## MATERIALS AND METHODS

### dCas9 fusion protein design and construction

The catalytic domain (CD) of human DNMT3A (amino acids 598 to 912 of NP_783328.1) both with and without the E756A mutation was cloned between the NheI and AgeI sites of pCMV_dCas9_VP64 (Addgene plasmid #49015, Cambridge, MA) with a NLS and FLAG tag linker. For lentivirus plasmids, TetO-FUW-OSKM (Addgene plasmid #20321), was digested with EcoRI and a multiple cloning site containing EcoRI, XbaI, NheI, AgeI, PspXI, AscI and EcoRI was cloned into this plasmid to generate TetO-FUW plasmid. An IRES-mCherry insert was amplified by PCR and cloned into AgeI- and AscI-digested TetO-FUW to generate TetO-FUW-IRES-mCherry. dCas9-D3A or dCas9-mD3A was cloned into this plasmid using XbaI and AgeI sites to generate TetO-dCas9-D3A or TetO-dCas9-mD3A. In the final plasmid, expression of both fusion proteins is controlled from the tetracycline operator (tetOP) and a minimal CMV promoter. The full amino acid sequence of the dCas9-3a or dCas9-3aΔ protein is shown in Fig. S4.1. Plasmids sequences were validated by Sanger sequencing and prepared for transfection using a Qiagen Maxiprep kit. All plasmids are available in Addgene, and detailed protocol information is available at http://epigenomics.wustl.edu/epigenomeEditing and http://www.challenlab.com.

### sgRNA design

Target sequences were entered into the MIT sgRNA design software (http://crispr.mit.edu/), the BROAD sgRNA design tool (http://www.broadinstitute.org/rnai/public/analysis-tools/sgrna-design-v1) (Doench et al., 2014), and the sgRNAcas9 tool (version 2.0.10) (Xie et al., 2014). The intersection of sgRNA target sites produced by all tools was taken for further analysis. sgRNA sequences that failed the BROAD test (score<0.2) were excluded. sgRNA were selected based on high BROAD scores and location relative to other sgRNAs. sgRNA coordinates and sequences are in Tables S1 and S2. Oligonucleotides corresponding to the target sites were annealed and cloned into MLM3636 (Addgene plasmid #43860). For lentivirus plasmids, U6-sgRNA coding sequences were amplified from pMLM3636 by PCR and cloned into an EcoRI and ClaI linearized pLVTHM lentiviral backbone (Addgene plasmid #12247) generating pL-sgRNA1 and pL-sgRNA2 in which GFP is expressed from EF1a promoter.

### Cell culture, lentiviral production and doxycycline induction

HEK293T cells were acquired from ATCC (CRL-3216, Manassas, VA) and grown in DMEM supplemented with 10% FBS (Gibco, Waltham, MA), 1× Penicillin/Streptomycin (Gibco), and 2 mM GlutaMax (Gibco). For transfection experiments, 3×10^5^ HEK293T cells were plated in a 60 mm dish. The next day, the cells were transfected with Lipofectamine LTX (Thermo Fisher Scientific, Waltham, MA). The Lipofectamine:DNA ratio was 3.5, with a total of 5.5 μg of plasmid DNA. The mass of Cas9-DNMT3A-CD fusion plasmid was equal to the total mass of the sgRNA plasmids. Since HEK293T cells incorporate either all plasmids or none, 0.5 or 0.7 μg of pMaxGFP was co-transfected in order to indicate the transfection efficiency. The plasmid DNA was first diluted in Gibco OptiMEM, then Lipofectamine LTX was added and mixed in by inversion. After 30 min, the transfection mixture was added dropwise to the cells and they were placed back in the incubator. 32D cells (ATCC) cells were maintained in RPMI supplemented with 10% FBS and 1% penicillin-streptomycin along with 5 ng/ml mouse interleukin-3 (IL-3). For lentiviral production, 293T cells were co-transfected with the packaging plasmids pMD2.G and psPAX2 along with the respective lentiviral plasmid (TetO- dCas9-3a, TetO-dCas9-3aΔ, pL-sgRNA1 and pL-sgRNA2). Viral supernatant was concentrated by centrifugation at 76,000 ***g*** for 1.5 h at 4°C. Cells were co-transduced with lentiviruses for the target gene expression along with an rtTA lentivirus (Addgene plasmid #20342) which is necessary for the activation of doxycycline controlled promoter in vectors containing the target gene. For transductions, a total of 1×10^5^ 32D cells were plated into 12-well plates with DMEM containing 10% fetal bovine serum and 1× penicillin/streptomycin in the presence IL-3, spin-infected with lentivirus at MOI of 1:1 at 250 ***g*** for 2 h. 24 h post-transduction, cells were washed and re-plated in fresh media for expansion. For the induction of fusion proteins in transduced 32D cells, 0.25 µg/ml doxycycline (Sigma, D9891-100G) was added to the growth media. Every other day, a half-media change with (0.5 µg/ml) doxycycline was performed.

### Sanger bisulfite sequencing

Genomic DNA (gDNA) was isolated using the PureLink Gemonic DNA Mini Kit (Invitrogen) or the Zymo Research (Irvine, CA) Quick gDNA MiniPrep kit and quantified with the Qubit dsDNA broad range assay (Thermo Fisher Scientific, Waltham, MA). gDNA was bisulfite converted with the Zymo Research EZ DNA methylation kit or the Epitech Bisulfite Kit (Qiagen) according to the manufacturer's instructions. All samples underwent bisulfite conversion with a high efficiency of at least 98% as determined by conversion of unmethylated, non-CpG cytosines. For *CDKN2A*, the target regions were amplified with the Qiagen PyroMark PCR kit and CDKN2A_B primers in Table S3. PCR products were cloned into the Promega pGEM-T Easy plasmid and transformed into NEB 10β competent cells. PCR products from individual colonies were sequenced by Sanger.

A nested PCR strategy was used to amplify *Cdkn1a* target genomic DNA site. In primary PCR reaction, 20 ng of total bisulfite converted DNA was used as a template with the primer pair F1 (GTTTAGATTTTTAGAGGGGAGGG) and R1 (CAAAAACTAAAAAAATAACTACCATCC). PCR was performed using Taq DNA polymerase (Invitrogen) using following cycling conditions; (1) 94°C for 3 min, (2) 95°C for 45 s, (3) 58°C for 58°C, (4) 72°C for 45 s and (5) 72°C for 10 min. Steps 2-4 were repeated for 40 cycles. For the second part of the nested PCR, 1.0 µl of the primary PCR reaction was used as a template and the same PCR conditions were used with primer pairs F1 and R2 (TCCTAAAATTCAAACTCTATATACC). The PCR amplicons were then gel extracted and cloned into pCR4-TOPO TA vector (Invitrogen) and resulting plasmids were transformed into TOP10 cells (Invitrogen). The target region was PCR amplified from different clones and the PCR products were sequenced by the Beckman Coulter Genomics (Danvers, MA, USA). Sanger bisulfite sequencing analysis was performed using BIQ Analyzer (Bock et al., 2005).

### Amplicon bisulfite sequencing

gDNA was extracted, bisulfite converted and PCR amplified as above. A tenfold molar excess of Illumina sequencing Y-adapters was then annealed to 100 ng of PyroMark PCR product (total Y-adapter mass varies with PCR product length) with NEB Quick Ligase for 15 min. The ligations were purified over a 1.5% agarose II (ISC BioExpress, Kaysville, UT) gel in 1× TBE in order to remove incompletely ligated DNA. Custom 13 bp barcode index sequences were added via PCR using NEB Phusion (NEB, Ipswich, MA). The standard Phusion PCR protocol was followed but with the following primer concentrations (for a 50 μl reaction): 1 μl of Illumina Primer 1.0 (25 μM), 1 μl of Illumina PCR Primer 2.0 (0.5 μM), and 1 μl of the index primer (25 μM). The thermocycler protocol was the following: (1) 98°C, 30 s; (2) 98°C, 10 s; (3) 64°C, 30 s; (4) 72°C, 30 s; (5) Return to step two 11 times; (6) 72°C 5 min; (7) 4°C hold. The PCR products were again purified over a 1.5% agarose II gel in 1× TBE, and their concentration was measured with the Qubit dsDNA high sensitivity kit. Individually indexed samples were pooled and submitted for sequencing.

Amplicon bisulfite sequencing data were checked for quality using fastQC, adaptor and poor quality sequence (quality less than 20) was trimmed using fqtrim, and the trimmed sequences were mapped to the target sequences using Bismark (Krueger and Andrews, 2011).

### ELISA methylation analysis

For quantification of total 5-mC level, the ELISA-based MethylFlash Methylated DNA Quantification Kit (Colorimetric; all from Epigentek, distributed by BioCat GmbH, Heidelberg, Germany) were utilized to quantify the amount of 5-mC in the DNA of transduced 32D cell lines. Quantification was performed in technical and biological duplicates according to the manufacturer's instructions. 100 ng of total DNA isolated from each transfected cells was used for 5-mC quantification. For absolute 5-mC quantifications, a standard curve was generated by plotting the various concentrations of the positive control provided with the kit against the optical density (OD) at 450 nm.

### Expression analysis

For human cells, RNA was extracted with the Zymo Research Quick mRNA Miniprep kit. RNA concentration was measured with the Qubit RNA BR kit. RNA integrity was determined by visualizing rRNA bands using agarose gel electrophoresis. Reverse transcription was performed using the Bio-Rad iScript Reverse Transcriptase kit. Quantitative reverse-transcription polymerase chain reaction (RT-qPCR) was performed with the Bio-Rad iTaq Universal with SYBR Green reagent on an Applied Biosystems Viia7 instrument. The thermocycler protocol was the following: (1) 95°C, 20 s; (2) 95°C, 3 s; (3) 60°C, 20 s; for 40 cycles. qPCR primers are listed in Table S4. A melt curve was performed to indicate there was not off-target amplification. Data was analyzed as described by Hellemans et al. (2007) using the geometric mean of *ACTB*, *GAPDH*, and *RPL0* as an internal control. The All sgRNA sample represents data from two independent transfection experiments. The data for all remaining samples derives from technical replication using the same RNA sample. *P*-values were calculated with paired sample *t*-tests on the normalized levels of gene expression.

For mouse cells, total RNA was isolated from 5×10^3^ FACS-sorted cells using NucleoSpin RNA XS (Macherey-Nagel) and reverse transcribed with the SuperScript VILO kit (Life Technologies). cDNA input was standardized and real-time polymerase chain reaction (PCR) was performed with TaqMan master Mix (Applied Biosystems), 18 s-rRNA probe (VIC-MGB; Applied Biosystems), and *Cdkn1a* gene probe (FAM-MGB; Mm04205640_g1, Applied Biosystems) on a StepOnePlus Real-Time PCR System (Life Technologies). Samples were normalized to expression of 18S and fold change determined by the ΔΔCt method.

### Western blot

32D cells transduced with dCas9-D3A or dCas9-mD3A with or without sgRNAs were grown in the presence of doxycycline for 12 days, collected and washed with ice cold PBS twice. Cells were then lysed in complete RIPA buffer containing protease inhibitors (Santa Cruz Biotechnology). 20 µg of protein lysates were separated on 10% SDS-PAGE gels and transferred to nitrocellulose membranes (Millipore). Membranes were subsequently probed to detect fusion proteins using primary antibodies recognizing Cas9 (Active Motif) or β-actin (Santa Cruz) and detection was performed using horseradish-peroxidase-conjugated secondary mouse antibody (Santa Cruz) and chemiluminescence (Millipore).
